# Body Stalk Anomaly Complicated by Ectopia Cordis: First-Trimester Diagnosis of Two Cases Using 2- and 3-Dimensional Sonography

**DOI:** 10.3390/jcm12051896

**Published:** 2023-02-27

**Authors:** Elisa Pappalardo, Ferdinando Antonio Gulino, Carla Ettore, Francesco Cannone, Giuseppe Ettore

**Affiliations:** Department of Obstetrics and Gynaecology, Azienda di Rilievo Nazionale e di Alta Specializzazione (ARNAS) Garibaldi Nesima, 95124 Catania, Italy

**Keywords:** body stalk anomaly, malformation, ultrasound, ectopia cordis

## Abstract

Introduction: Body stalk anomaly is a severe defect of the abdominal wall, characterized by the evisceration of abdominal organs and, in more severe cases, thoracic organs as well. The most serious condition in a body stalk anomaly may be complicated by ectopia cordis, an abnormal location of the heart outside the thorax. The aim of this scientific work is to describe our experience with the prenatal diagnosis of ectopia cordis as part of the first-trimester sonographic screening for aneuploidy. Methods: We report two cases of body stalk anomalies complicated by ectopia cordis. The first case was identified during a first ultrasound examination at 9 weeks of gestation. The second was identified during an ultrasound examination at 13 weeks of gestation. Both of these cases were diagnosed using high-quality 2- and 3-dimensional ultrasonographic images obtained by the Realistic Vue and Crystal Vue techniques. The chorionic villus sampling showed that the fetal karyotype and CGH-array were both normal. Results: In our clinical case reports, the patients, immediately after the diagnosis of a body stalk anomaly complicated by ectopia cordis, opted for the termination of pregnancies. Conclusion: Performing an early diagnosis of a body stalk anomaly that is complicated by ectopia cordis is desirable, considering their poor prognoses. Most of the reported cases in the literature suggest that an early diagnosis can be made between 10 and 14 weeks of gestation. A combination of 2- and 3-dimensional sonography could allow an early diagnosis of body stalk anomalies complicated by ectopia cordis, particularly using new ultrasonographic techniques, the Realistic Vue and the Crystal Vue.

## 1. Introduction

Body stalk anomaly is a congenital abnormality of the abdominal wall, depending on the evisceration of abdominal organs and, in more complicated clinical cases, thoracic organs. This anomaly is usually characterized also by kyphoscoliosis and by a defect of the umbilical cord, which is usually short or not present [1]. This pathological condition could also be related to neural tube anomalies, genital and urinary abnormalities, chest wall defects, bowel atresia, and craniofacial defects. The most serious condition in a body stalk anomaly may be complicated by ectopia cordis, an abnormal location of the heart outside the thorax. The wide number of phenotypes reported in the scientific literature has allowed the use of different medical terms to define this congenital pathology, such as “amniotic band syndrome” or “short umbilical cord syndrome” [2]. 

The possible causes of a body stalk anomaly include early amnion rupture with direct mechanical pressure and amniotic bands, vascular disruption of the early embryo, or an abnormality in the germinal disk.

Body stalk anomaly is a rare congenital defect with an incidence rate of 1 in 14,000 to 1 in 31,000 pregnancies, according to large epidemiologic data. In a scientific work published in the last few years, based on the evaluation of 106,727 fetuses between 10 and 14 weeks of gestational age, the rate is approximately 1/7500 pregnancies, considering the elevated incidence of miscarriages related to this condition [3,4].

Although in the scientific literature there are many works on body stalk anomalies with a diagnosis performed in an advanced stage of pregnancy, studies about diagnosis between 10 and 14 weeks of gestational age are still few [5,6]. This study was based on the evaluation of the sonographic characteristics of this infrequent abnormality in the first three months of pregnancy using 2-dimensional (2D) and 3-dimensional (3D) sonography. We present the prenatal diagnosis and management of two cases of body stalk anomaly complicated by ectopia cordis, large abdominal wall defects, limb deformities, kyphoscoliosis, spina bifida, and acrania in the first trimester: the first case at 10 weeks of gestation and the second at 13 weeks of gestation.

The purpose of this scientific work is to describe our clinical experience with the prenatal diagnosis of ectopia cordis in routine sonographic screening performed in the first three months of pregnancy for the risk of chromosomal abnormalities. The description of the case reports was performed following the CARE criteria (https://www.care-statement.org/checklist (accessed on 30 December 2022). The study protocol was approved by the Ethics Committee of the ARNAS Garibaldi Hospital and conformed to the ethical guidelines of the Helsinki Declaration. The women signed informed consent before entering the study, and their anonymity was preserved. 

The ultrasonographic images and video were obtained by a Samsung Hera W10 ultrasound, and the high-quality 2D and 3D images were obtained by Realistic Vue and Crystal Vue techniques. 

REALISTIC VUE allows a high-resolution 3D visualization of anatomical details with extremely realistic depth perception. The direction of the light source can be selected by the operator, creating graduated shadows that allow better definition of the different anatomical structures.

CRYSTAL VUE is a 3D rendering software that helps locate fetal morphological anomalies through intuitive visualization of internal and external structures. Its diffusion is due to the progress made in terms of the quality of images, technologies, and ease of use. Crystal Vue is one of the most modern volumetric rendering technologies that retains the context and surface information of 3D ultrasound. This new method facilitates differentiation between soft tissue contours and anatomical structures with automatic settings in order to obtain optimal images in complex situations. Crystal Vue also displays the internal and external structures and provides additional information to enable detailed anatomical evaluation and the diagnosis of anomalies. It can also be activated in combination with the color doppler mode.

## 2. Cases Description

### 2.1. Case Report 1

A 25-year-old woman was referred for an ultrasound scan at 9 weeks of gestation.

There was no relevant medical history, and she was taking no medication. There were no teratogenic risk factors in the clinical history of the woman. It was her second pregnancy, and her first pregnancy was uneventful. The ultrasound scan showed a normal fetal crown-rump length and a not-normal site of the inferior part of the embryo in the coelomic cavity, and there were multiple fetal abnormalities. The abnormal findings were an anterior thoracoabdominal wall defect containing liver and bowel, ectopia cordis, severe kyphoscoliosis, deformed lower limbs, a spina bifida, and an acrania with exencephaly. All these findings were compatible with the diagnosis of a body stalk anomaly complicated by ectopia cordis. The umbilical cord was also located extra-amniotically but had a normal length. 

**Figure 1:**
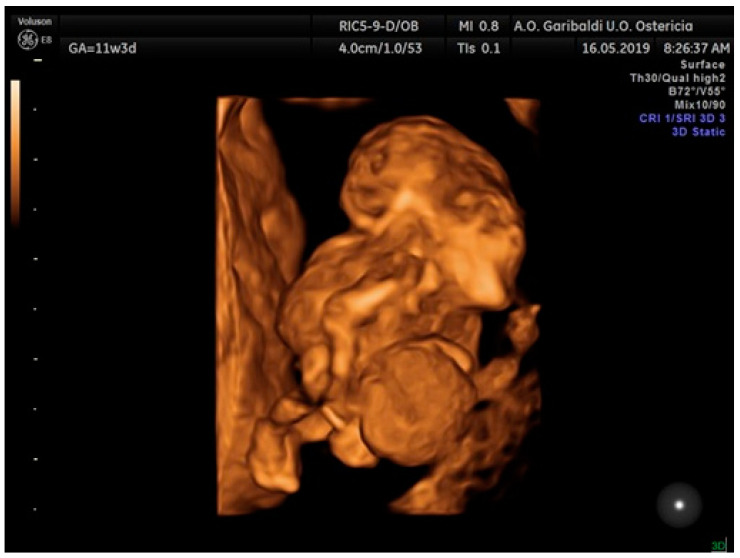
3D Image of case report 1 at 11 weeks.

Realistic Vue and Crystal Vue ultrasonographic techniques allow an early diagnosis of this pathological condition at 9 weeks of gestation, but to confirm this diagnosis, the woman was scheduled for a second ultrasound scan at 11 weeks of gestation (Figure 1). Unfortunately, we do not have any images of the first ultrasound scan, performed at 9 weeks of gestation, but only images of the second ultrasound scan. The ultrasound findings at 11 weeks of gestation confirmed the abnormal aspects evaluated at 9 weeks. The Realistic Vue and Crystal Vue ultrasonographic techniques were extremely useful to achieve this diagnostic objective because they allowed an accurate visualization of internal and external structures of the thoracoabdominal wall.

Chorionic villus sampling showed that the fetal karyotype and CGH-array were both normal. Considering that this malformation was not compatible with life, the patient decided to plan the termination of her pregnancy. 

**Figure 2:**
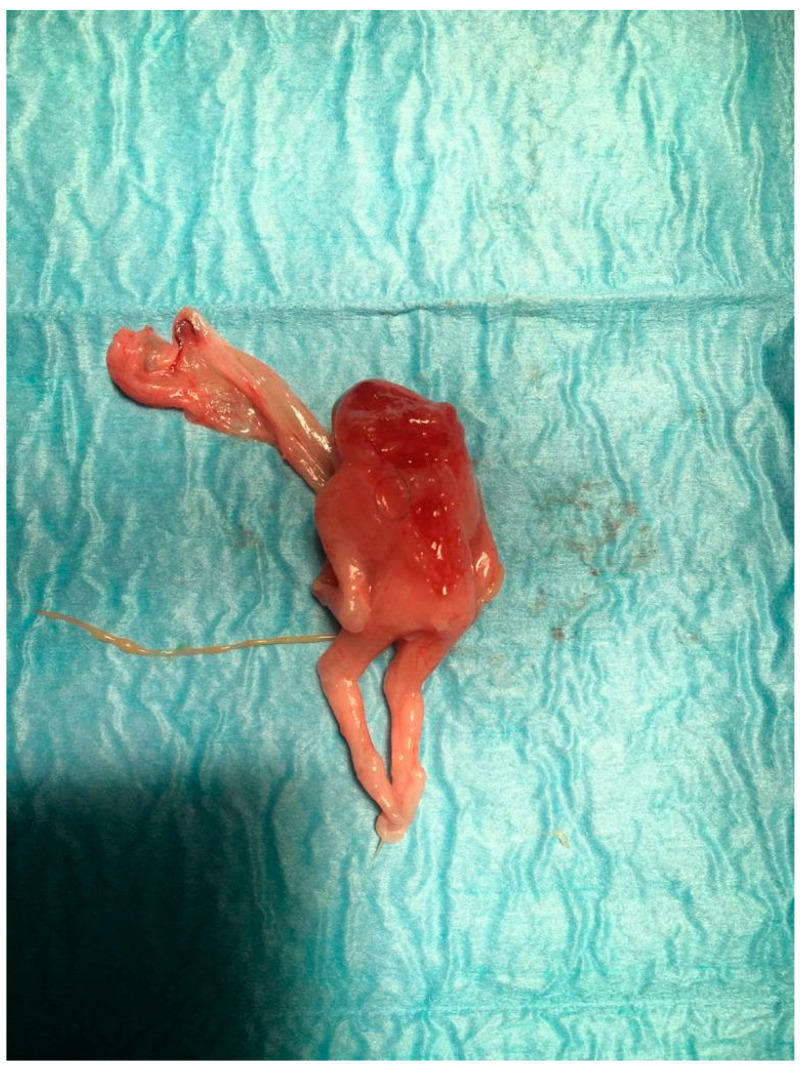
Macroscopic pathological examination of case report 1 at 11 weeks.

After the procedure, the embryo and the placenta were sent to the department of pathology, which showed these morphologic characteristics: a non-normal cephalic extremity with acrania and rachischisis, regular upper limbs, no presence of the right leg, the left leg bent toward the chest, and an abnormal omphalocele in which there was the presence of the bowels and liver. The placenta measured 5 × 4 cm. The umbilical cord was positioned anteriorly, characterized by a central insertion and a short length of 2 cm and 0.3 cm diameter, wrapped by amniotic membranes that trapped the fetus. The fetus was affected by a severe malformation and showed the presence of ectopia cordis, with a heart outside the thorax. There was an important anomaly of the anterior and lower thoracic walls and the abdominal wall, with an external presence of the bowel, spleen, and liver (Figure 2). 

### 2.2. Case Report 2

A 43-year-old woman was referred for routine sonographic screening for chromosomic abnormalities at 13 weeks of gestational age. It was her third pregnancy, and her previous pregnancies were uneventful. There was no relevant medical history, and she was taking no other medication. 

**Figure 3:**
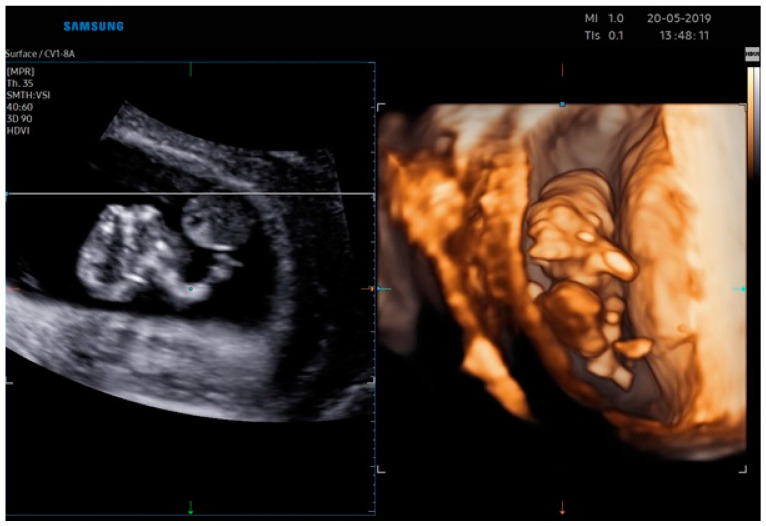
Realistic Vue image of case report 2 at 13 weeks.

**Figure 4:**
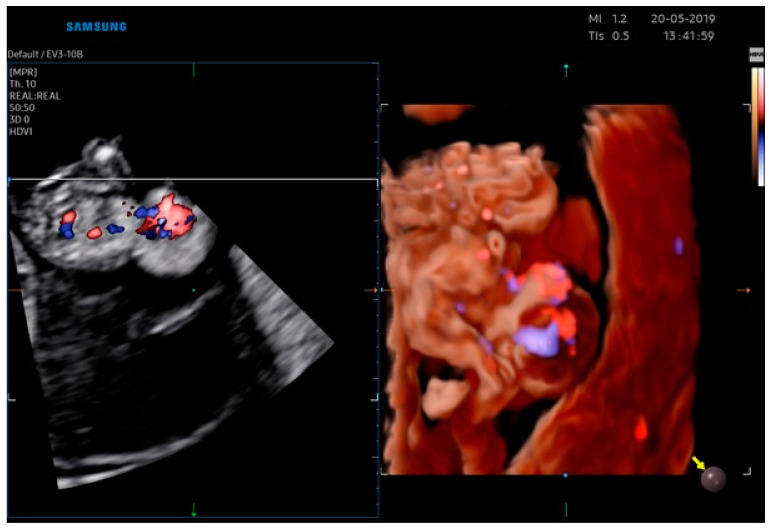
Realistic Vue image of case report 2 at 13 weeks.

**Figure 5:**
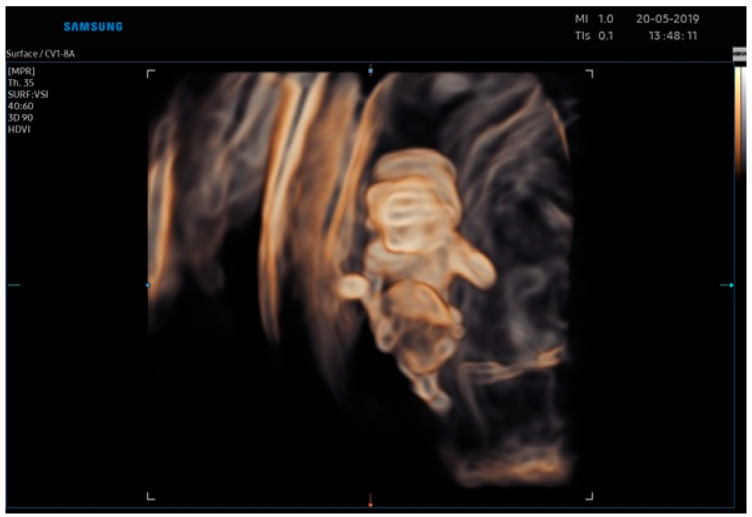
Crystal Vue image of case report 2 at 13 weeks.

The ultrasonographic findings by Realistic Vue image (Figure 3 and Figure 4) and Crystal Vue image (Figure 5) at 13 weeks (Supplementary File: Video S1) showed a regular crown-rump length (CRL) of 4 cm and multiple fetal abnormalities. The combination of defects was represented by a large skull and brain defect, an anterior thoracoabdominal wall defect, a heart pulsating outside the thorax, severe kyphoscoliosis and deformed lower limbs, a liver directly attached to the placenta without an interposed umbilical cord, increased distortion of the spine, a separation between the celomatic and amniotic cavities, and a short umbilical cord. All these abnormalities were compatible with the diagnosis of a body stalk anomaly. 

The chorionic villus sampling showed that the fetal karyotype and CGH-array were both normal.

Considering that this diagnosis was considered to be incompatible with life, the patient decided to proceed with a termination of pregnancy. 

**Figure 6:**
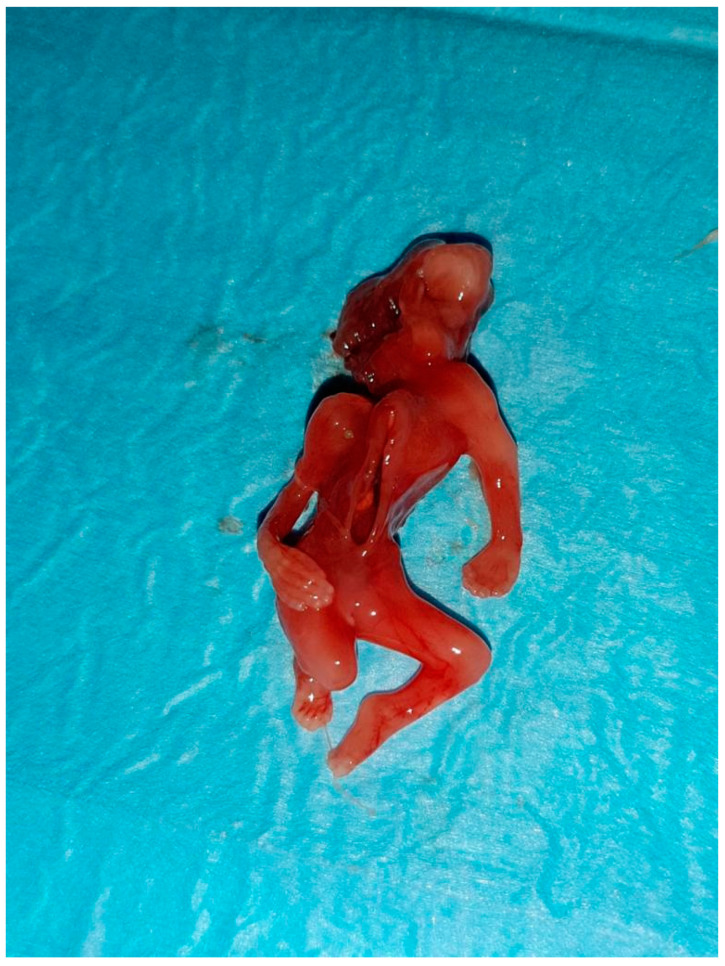
Macroscopic pathological examination of case report 2 at 13 weeks.

The embryo and the placenta were sent to pathology; the embryo showed exencephaly, severe kyphoscoliosis, increased distortion of the spine, defects of the neural tube, ectopia cordis, and deformed lower and upper limbs (Figure 6).

## 3. Discussion

In our scientific work, we described our experience with two cases of prenatal diagnosis of a body stalk anomaly complicated by ectopia cordis during a routine sonographic screening for chromosomic abnormalities in the first trimester. A body stalk anomaly is defined as a pathological congenital condition of multiple abnormalities, which are, in most of the reported scientific cases, not compatible with life. As described above, this condition should be suspected when a large abdominal defect is observed and is associated with other abnormalities in the axial skeleton, such as kyphosis or scoliosis, or a short or absent umbilical cord. Body stalk defects could be detected by ultrasound at the end of the first trimester of pregnancy. 

This congenital syndrome has an incidence rate of 1/14,000 to 1/31,000 pregnancies. In a recent multicenter scientific study performed by Daskalakis et al. [3] there was a rate of 1/7500 pregnancies. This big difference in the incidence rates could be related to the high rate of miscarriages associated with body stalk anomalies, which could not allow a diagnosis during the first trimester of pregnancy; therefore, the exact incidence rate might be underestimated [6,7,8,9,10,11,12].

Van Allen et al. described this syndrome for the first time in 1987 [1], and they mentioned three essential features:

- Exencephaly, or facial clefts, or encephalocele;
- Thoraco or abdominoschisis;
- Limb defects.

In the study of Van Allen et al. “body stalk anomaly” is defined as a defect of embryonic blood flow in the first phases of embryonic development, between 4 and 6 gestational weeks. This condition determines a defect in the ventral wall’s closure and maintains the coelomic cavity’s persistence. The clinical evaluation that found cocaine abuse could raise the risk of a body stalk anomaly confirms this etiopathogenetic theory of vascular impairment [13]. Furthermore, another study proposed as an etiopathogenetic defect an early failure of fetal folding along the cephalic, lateral, and caudal axes [4]. In the scientific literature, body stalk anomaly is represented as two distinct phenotypes: the “placenta-cranial” phenotype, where cranial abnormalities and cranio-placental attachment are the main signs, and the “placenta-abdominal” phenotype, where the lower part of the fetus is sited within the not-obliterated extraembryonic cavity [14]. Our case reports describe placenta-abdominal phenotypes.

The actual mechanism, however, remains unclear. In most of the described cases, the karyotypes of the affected fetuses have been completely normal, and only in two cases have there been chromosomal abnormalities associated with uniparental disomy of chromosome 16 and with a trisomy of chromosome 2 [15]. This is probably due to confined placental mosaicism. Hence, what is known about the defects of the body stalk is that environmental and genetic factors play an important role in the pathophysiology of this complex and poorly understood condition.

In a review of 11 cases of body stalk anomaly by Smrcek et al. [4], four cases (36%) were complicated by ectopia cordis. On the other hand, two (50%) of four cases of ectopia cordis were associated with a body stalk anomaly, as was seen in a review by Sepulveda et al. [5]. Ectopia cordis is another pathological congenital condition with a complete or partial shift of the heart outside the thorax. The etiopathology of ectopia cordis is represented by a stop in the heart’s infolding, which normally occurs after four weeks of pregnancy [14]. Ectopia cordis is subdivided into four types following the site of the heart [14]:

- Cervical (3% of cases);
- Thoracic (60% of cases);
- Thoracoabdominal (7% of cases);
- Abdominal (30% of cases).

The outcome of this condition is unfavorable due to intracardiac and extracardiac structural anomalies. Although usually the diagnosis is easy and clear and could be made at 10 weeks of gestational age, cases of thoraco-abdominal ectopia cordis could be difficult to diagnose if only the apex of the heart is extra-thoracic, and visualization is hindered by extruded abdominal organs [16,17,18,19,20].

Considering the increasing quality of first trimester ultrasonographic evaluation as a screening tool for aneuploidy in current clinical practice, it is reasonable to expect that most cases of ectopia cordis will be diagnosed at an early gestational age. For this reason, several cases describing the diagnosis before 14 weeks of gestational age have been reported in the scientific literature, but most of them are only isolated case reports [21,22,23,24,25].

An appropriate ultrasound scan of the first trimester with the right measurement of the crown-rump length should make a diagnosis of all cases of body stalk anomaly between 11 and 13 weeks of pregnancy [22]. A final diagnosis should exclude the following differential congenital pathological conditions: omphalocele, gastroschisis, vesical exstrophy, Cantrell pentalogy, amniotic band syndrome, Beckwith-Wiedemann syndrome, and the OEIS complex.

In our scientific work, we did not find any signs that could be related to environmental exposure to teratogens. However, in some case series described in scientific literature, it has been demonstrated that 50% of pregnant women with a fetal diagnosis of a body stalk anomaly smoke cigarettes or drink alcohol, and 30% of them smoke marijuana [24]. In our clinical cases, we also found that the fetal karyotype and CGH-18 array were both normal; these findings confirmed that the pathogenetic basis of these syndromes is not genetically based but rather a disruption. 

The advantages of 3D ultrasound are its multiplanar and surface-rendering modalities. The multiplanar view helps the sonographer better correlate the anomaly in the three orthogonal planes simultaneously, providing more details. Then, the surface mode could provide a “sculpture-like” picture that could be rotated in all directions, allowing inspection from different angles. In this study, 2D ultrasound combined with 3D ultrasound showed more detailed features than 2D ultrasound alone. 

The strength of our work is the description of two rare cases of body stalk anomaly complicated by ectopia cordis using new ultrasonographic techniques, the REALISTIC VUE and the CRYSTAL VUE, which could allow diagnosis of these malformations in an early stage of pregnancy with high accuracy. The knowledge of body stalk anomaly aetiology may guide the sonographer to carry out a diagnosis at an early stage and to eventually prevent a recurrence. 

The main limitation of our work is related to the descriptions of only two case reports; in the scientific literature, the main limitation of this pathological condition is represented by the heterogeneity of the description of its features, based mainly on the report of single case reports. Further research on this topic is needed.

## 4. Conclusions

Body stalk anomaly is a congenital pathological condition with uncertain etiopathogenesis, uncertain pathophysiology, and an uncertain incidence rate. In the scientific literature, most of the reported clinical cases described an early diagnosis performed between 10 and 14 weeks of gestation; in our first case report, the diagnosis of a body stalk anomaly complicated by ectopia cordis was suspected earlier, at nine weeks of pregnancy, thanks to the use of 2- and 3-dimensional Sonography, particularly by new ultrasonographic techniques, the Realistic Vue and the Crystal Vue. This could represent an important turning point in the diagnosis of this pathological congenital syndrome because it could allow knowing the fetus’s condition early and giving appropriate counseling to the parents and prompt management to the physicians.

## Data Availability

Data is contained within the article.

## Supplementary Materials

The following supporting information can be downloaded at: https://www.mdpi.com/article/10.3390/jcm12051896/s1, Video S1. (Body stalk anomaly by Crystal Vue sonographic imaging).
